# Screening of Basidiomycete Strains Capable of Synthesizing Antibacterial and Antifungal Metabolites

**DOI:** 10.3390/ijms26199802

**Published:** 2025-10-08

**Authors:** Valeria Lysakova, Aleksey Streletskiy, Olga Sineva, Elena Isakova, Larissa Krasnopolskaya

**Affiliations:** 1 Gause Institute of New Antibiotics, Bol’shaya Pirogovskaya Str., 11, 119021 Moscow, Russia; lisakova2000@gmail.com (V.L.);; 2 Centre for Strategic Planning of FMBA of the Russian Federation, Pogodinskaya St., Bld.10, 119121 Moscow, Russia

**Keywords:** basidiomycetes, submerged cultivation, extracts, antibacterial activity, antifungal activity, HPLC-MS

## Abstract

Recently, the search for new antimicrobial compounds, including the secondary metabolites of basidiomycetes, has become increasingly important. Representatives of this division of higher fungi have high biosynthetic abilities, which contributes to their use as producers. In this work, extracts of culture liquids and submerged mycelia from 18 strains representing three different orders of basidiomycetes were studied. For this purpose, the submerged cultivation of strains, extraction of biological material, and evaluation of the extract’s antimicrobial activity using the agar well diffusion method were carried out. The minimum inhibitory concentration was determined for extracts with strong activity. The most promising ones were analyzed using HPLC-MS. As a result, it was found that 16 strains contained antimicrobial metabolites. Thus, the strains selected for further work were *Hericium corraloides* 4, which showed not only the antibacterial but also antifungal activity of cultural liquid and submerged mycelia extracts, and *Fomitopsis betulina* 3, *Fomitopsis pinicola* 2, *Hericium erinaceus* 1, and *Laetiporus sulphureus* 4, whose cultural liquid extracts exhibited high antibacterial activity against Gram-positive and Gram-negative test cultures. For these strains, metabolic profiles were obtained using the method HPLC-MS. Using this method, two metabolites were preliminary identified: hericerin in *H. erinaceus* 1 and sulfureuine H in *L. sulphureus* 4.

## 1. Introduction

The occurrence of resistant strains of pathogenic microorganisms is increasing every year [1]. There are many reasons for this emergence, such as the uncontrolled use of antibiotics, the discharge of chemical waste into the environment, etc. [2,3]. Another reason is the narrow range of available antibiotics. Therefore, it is an important task to identify and isolate new antimicrobial molecules, in particular, from natural sources. This approach was also used at the beginning of the Antibiotic Era in the 20th century, when there was an active search for new producers of antimicrobial molecules. Actinomycetes and ascomycetes were often used as such producers, which are still the main groups of producers [4,5]. In our work, we focused on a less studied group of producers, basidiomycetes.

Basidiomycetes are one of the most important divisions of higher fungi. According to the latest data, this division includes from 0.7 to 1 million species, which is about 28–40% of the total fungal diversity [6,7]. They are common in all geographical areas, growing on various substrates. Basidiomycetes are mainly saprotrophs: they play an important role in the organic compound’s mineralization, releasing their enzymes into the environment and absorbing the decomposition products of organic compounds. In addition, some of them may also be symbionts or parasites of various organisms [6].

Basidiomycetes have a rich and diverse secondary metabolism. Their secondary metabolome contains unique features, as well as similarities with the metabolome of other fungi and plants, primarily among polypeptides, acetylenes, and sesquiterpenoids [8]. The secondary metabolites of basidiomycetes differ significantly in their biogenetic origin, physiological role, structure, and application. These substances are now the subject of many studies of fungi. Scientists have discovered antioxidants, antimicrobial compounds, and antitumor, anti-inflammatory, and other molecules in the composition of metabolites [9,10].

Although medicinal fungi have been used by various cultures for centuries, they only recently have been researched for use in modern medicine. For example, medicinal fungi-based products are used as an adjunct in the treatment of cancer, alleviating the side effects of chemotherapy and radiotherapy, such as nausea, anemia, and immunity decreased [11].

Many medicinal fungi are edible—in addition to biologically active metabolites with beneficial properties, they contain all essential amino acids, as well as vitamins: thiamine, riboflavin, nicotinic acid, and ascorbic acid. Medicinal and edible fungi are rich in minerals such as calcium, phosphorus, iron, sodium, and potassium. That is why fungi can be considered as a rational dietary supplement and also as a means for the prevention (and treatment) of diseases [12].

Antimicrobial properties of basidiomycetes have been studied since the 1940s [13]. These studies are now becoming more intensive [10]. Most screening studies on this topic are devoted to the study of the antimicrobial activity of fruit bodies collected in nature, while there are significantly fewer studies involving submerged cultivation [10]. Most metabolites with targeted properties are detected and isolated from the culture liquids [14,15]. Therefore, it is rational to conduct a search, initially using the submerged cultivation of the tested strains.

Antimicrobial metabolites isolated from basidiomycetes belong to various chemical classes of compounds—terpenoids, sesqiterpenes, lactones, carboxylic acids, peptides, and other various compounds. They have activity against various microorganisms. These can be Gram-positive [16,17,18] and Gram-negative [19,20] bacteria, including clinical and resistant strains [21,22], fungi [15,23], and phytopathogens [24]. It should be noted that activity against Gram-negative bacteria is much less common than against Gram-positive bacteria [10].

Since there is insufficient information on the antimicrobial properties for many species of basidiomycetes, further careful study is necessary, starting with testing their extracts. In our work, for the reproducibility of the results, we carried out the cultivation in a submerged culture.

The aim of the study was to evaluate the antimicrobial activity of 18 submerged cultures of basidiomycetes belonging to the orders of Agaricales, Polyporales, and Russulales, and to select the most promising strains producing antimicrobial metabolites and to conduct a preliminary assessment of the metabolic profile.

## 2. Results

### 2.1. Submerged Cultivation

In this work, 18 strains from the collection of basidiomycetes from the Laboratory of Biologically Active Compounds Biosynthesis at the Gause Institute of New Antibiotics were studied. All investigated strains belong to the same ecological and trophic group, xylotrophic basidiomycetes. At first, the process of basidiomycete biomass accumulation under submerged culture conditions was studied. The length of the submerged cultivation, 5–7 days, was chosen based on previous studies [25,26]. For each strain, the day with the maximum yield of submerged biomass was determined as an indicator of secondary metabolite release. These metabolites are known to include antibiotics. The results are presented in Table 1.

Most strains were characterized by pH values of the culture liquid in the range of 4–5; however, some strains of the Polyporales order acidified the medium to pH values of 1.5–2.5, which could indicate the presence of a large amount of acids among the metabolites of *Fomes fomentarius* 1, *Fomitopsis betulina* 3, *F. pinicola* 2, and *Laetiporus sulphureus* 4 strains. The most productive strain in terms of the production of submerged mycelia was *Pleurotus pulmonarius* 1. The fastest-growing were representatives of the species *Hypsizigus* and *Pleurotus*.

### 2.2. Determination of Antimicrobial Activity by Agar Well Diffusion Method

As a result of submerged cultivation, culture liquids and submerged mycelia were obtained for each of the studied strains to obtain extracts. Accordingly, a total of 36 extracts were studied. Dry extract samples were diluted to a concentration of 10 mg/mL and their antimicrobial activity was evaluated by the agar well diffusion method. The results are presented separately for each order in separate tables.

The studied extracts of the culture liquids and submerged mycelia of representatives of the Agaricales order, as a rule, did not exhibit high antibacterial and antifungal activity (Table 2). Extracts of the *Armillaria borealis* 1 and *Pleurotus ostreatus* 10 culture liquids had low activity against Gram-positive bacteria; all culture liquid extracts, with the exception of *P. pulmonarius* 1, showed weak antifungal activity against *Fusarium oxysporum*. Only the extract of the *A. borealis* 1 culture liquid showed activity against *Candida albicans*. Extracts of the submerged mycelia of representatives of the Agaricales order had no antimicrobial activity.

Representatives of the Polyporales order have demonstrated high antibacterial activity (Table 3). All the studied culture liquid extracts showed antimicrobial activity, which can be considered high, with the exception of the low activity of the *Grifola frondosa* 9 and *Trametes versicolor* 1 extracts.

**Figure 1:**
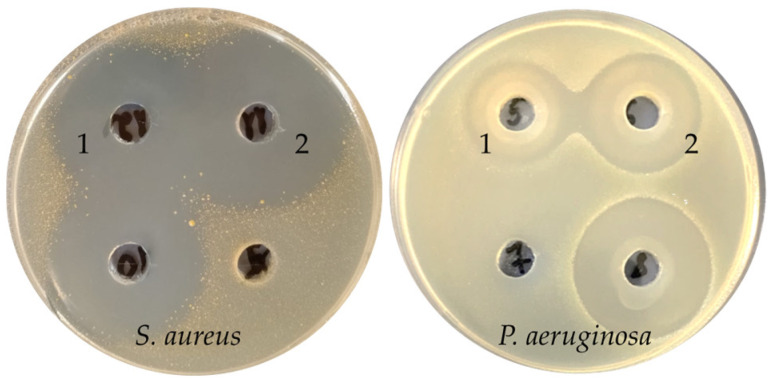
Antibacterial activity of *F. betulina* 3 (1) and *F. pinicola* 2 (2) extract culture liquids against *S. aureus* and *P. aeruginosa*.

Extracts of the *F. fomentarius* 1, *F. betulina* 3, and *F. pinicola* 2 (Figure 1) culture liquids were highly active against both Gram-positive and Gram-negative bacteria with inhibition zone diameters above 20 mm. The *F. betulina* 3 and *F. pinicola* 2 culture liquid extracts also demonstrated low antifungal activity against *F. oxysporum*, so these extracts showed the highest spectrum of activity among samples from the Polyporales order. The extract of culture liquid *L. sulphureus* 4 was highly active against Gram-positive bacteria, but activity against *Pseudomonas aeruginosa* and *F. oxysporum* was absent. Among the extracts of the submerged mycelia, extracts of *F. fomentarius* 1, *F. pinicola* 2, and *F. betulina* 3 were promising for the further isolation of antimicrobial metabolites, because they demonstrated activity against both Gram-positive and Gram-negative bacteria.

All the studied strains of the Russalales order, with the exception of *Hericium erinaceus* 9, were capable of producing antimicrobial metabolites (Table 4), which mainly accumulated in the culture liquid. Extracts of the culture liquids of these strains were highly active (inhibition zones 20+ mm) against Gram-positive bacteria, while only extracts of *Hericium coralloides* 4 and *H. coralloides* 18 were active against *P. aeruginosa*. Four of the six culture liquid extracts had antifungal activity, but the activity was only against mycelial fungi.

**Figure 2:**
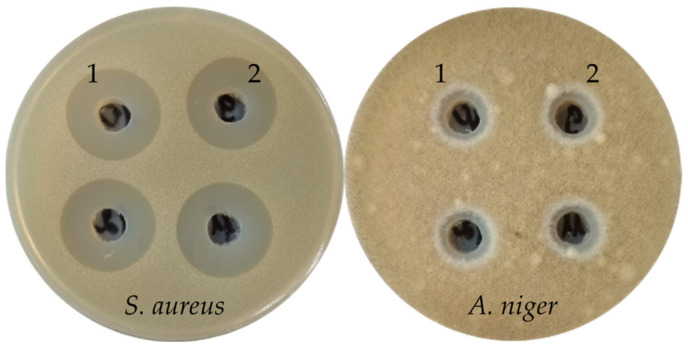
Antimicrobial activity of *H. coralloides* 4 extracts (1—extract of culture liquid, and 2—extract of submerged mycelium) against *S. aureus* and *A. niger*.

The extract of the *H. coralloides* 4 (Figure 2) submerged mycelium alone exhibited high antibacterial activity against Gram-positive bacteria (inhibition zones 21+ mm), and antifungal activity against mycelial fungi. All other submerged mycelia extracts from the strains of this order had no antimicrobial activity. In general, the extracts of *H. coralloides* strains were more active than the extracts of *H. erinaceus* strains.

### 2.3. Determination of Extracts’ Minimum Inhibitory Concentrations (MICs)

According to the results of the evaluation of the antimicrobial activity of extracts by the agar well diffusion method, the most promising producers of target metabolites were *F. fomentarius* 1, *F. betulina* 3, *F. pinicola* 2, *L. sulphureus* 4, *H. coralloides* 4, and *H. erinaceus* 1. The minimum inhibitory concentration (MIC) values for their extracts were determined on a panel of Gram-positive and Gram-negative bacteria. The obtained results are shown in Table 5. The values in the three repeats did not have significant differences, so single values are presented.

According to Kuete [27], the MIC values of extracts can be divided into three groups. Significant activity was MIC < 100 µg/mL, average activity was 100 < MIC < 625 µg/mL, and weak activity was MIC > 625 µg/mL. Kuete used this classification for plant extracts. Since basidiomycete extracts were of natural origin, this approach was used with a modification of the average activity range 160 > MIC > 640 µg/mL, due to the multiplicity of dilutions used in our work.

The most active was the extract of culture liquid *H. coralloides* 4, which showed high MIC values of 80 µg/mL against *Staphylococcus aureus* ATCC 25923 and 160 µg/mL against clinical strains *Staphylococcus epidermidis* and *Staphylococcus haemoliticus*, and 320 µg/mL against the clinical strain *S. aureus* 10 and the clinical strain *Enterococcus faecium* 569, resistant to vancomycin A.

Extracts of the *L. sulphureus* 4 and *H. erinaceus* 1 culture liquids were also promising. The former had an MIC of 320 µg/mL against all test cultures from the genus Staphylococcus, as well as an MIC of 640 µg/mL against *E. faecium*, *Acinetobacter baumanii,* and *Proteus vulgaris*. The extract of the *H. erinaceus* 1 culture liquid had moderate antimicrobial activity against Gram-positive bacteria *S. epidermidis* and *S. haemoliticus,* and Gram-negative bacterium *A. baumanii* (MIC 320 µg/mL). With respect to the remaining test cultures, this extract had an MIC of 640 µg/mL.

*Fomitopsis* species extracts had weaker antimicrobial activity. However, they had an MIC of 640 µg/mL against most of the test cultures used, unlike extracts of *H. coralloides* 4 and *L. sulphureus* 4. This indicates that the spectrum of activity of the *F. betulina* 3 and *F. pinicola* 2 metabolites includes both Gram-positive and Gram-negative bacteria, without selectivity of action. The *F. fomentarius* 1 culture liquid extract had weak activity, so it was not used in further studies.

### 2.4. Sequencing Results

The taxonomic identity of the selected five strains producing antimicrobial metabolites was confirmed by the Sanger sequencing of the ITS1–5.8S–ITS2 ribosomal DNA region. The obtained sequences were compared with the NCBI GenBank database using the BLAST version 2.1.2 algorithm. The coefficient of similarity between the obtained and reference sequences for species identification was ≥99.5%.

Strain *F. betulina* 3 demonstrated a 99.53% similarity to the voucher specimen Cui 17121 (GenBank accession OL621853.1). *F. pinicola* 2 demonstrated a 99.58% similarity to the isolate Bre_P3_13800209 (GenBank accession PP409503.1). *H. corallodes* 4 demonstrated a 99.84% similarity to the voucher specimen LE-BIN 1891 (GenBank accession MG735348.1). *H. erinaceus* 1 demonstrated a 99.72% similarity to the voucher specimen He-1 (GenBank accession FJ882088.1).

### 2.5. Extracts’ High-Performance Liquid Chromatography–Mass Spectrometry (HPLC-MS)

**Figure 3:**
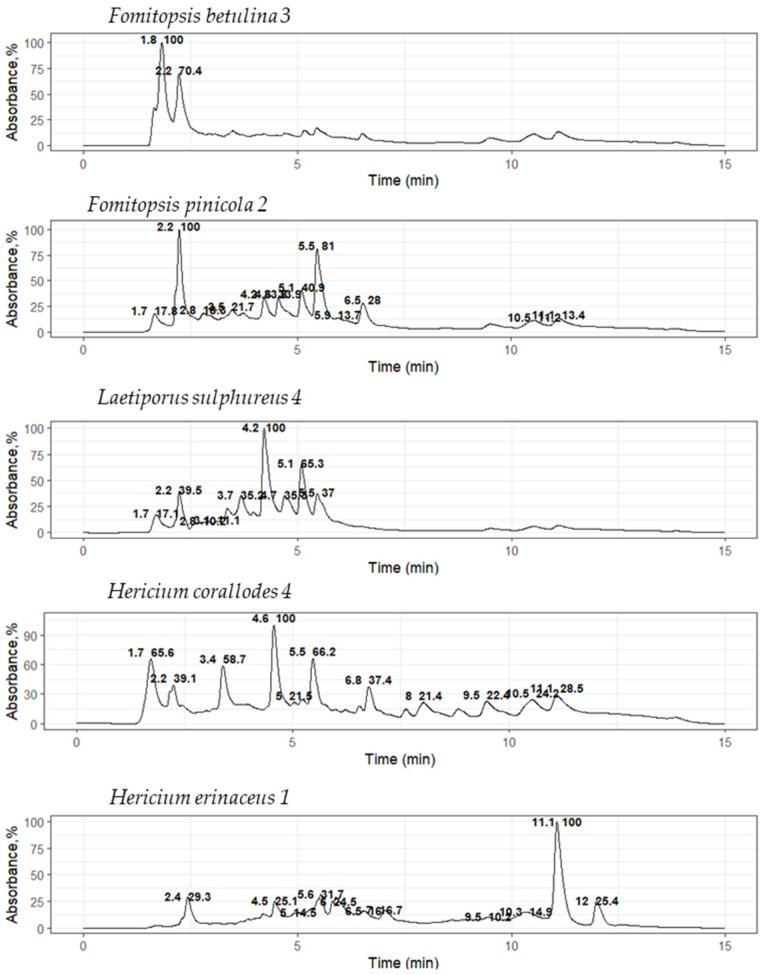
UV chromatograms of basidiomycete culture liquid extracts at a 280 nm absorption length.

It is known that basidiomycetes from different taxonomic groups can produce the same metabolites, for example, lovastatin, an inhibitor of HMG-CoA reductase, which has been found in such basidiomycetes as *P. ostreatus*, *Omphalotus olearius*, *T. versicolor*, *H. erinaceus*, *Coprinus comatus*, and others [28,29,30,31]. Therefore, it is important to identify the similarities of the metabolic profiles of the active extracts, which will then be used to isolate individual components. For this purpose, an HPLC-MS analysis was performed to evaluate and compare the metabolic composition of the active extracts. Extracts were selected based on both their MIC values and producer taxonomic position. Furthermore, preliminary similarities in metabolic composition were identified within the same genus. Chromatograms for extracts of the culture liquids *F. betulina* 3, *F. pinicola* 2, *H. coralloides* 4, *H. erinaceus* 1, and *L. sulphureus* 4 are shown in Figure 3.

The most intense ions (*m*/*z*) were determined from the mass spectrum recorded at the retention time (RT) of the peaks. For each such ion, an extracted ion chromatogram (EIC) was constructed and its compliance with the chromatographic peak detected by UV absorption or fluorescence was checked. The Table 6 shows the most relevant data, including the *m*/*z* of the most intense peaks with low noise levels.

**Figure 4:**
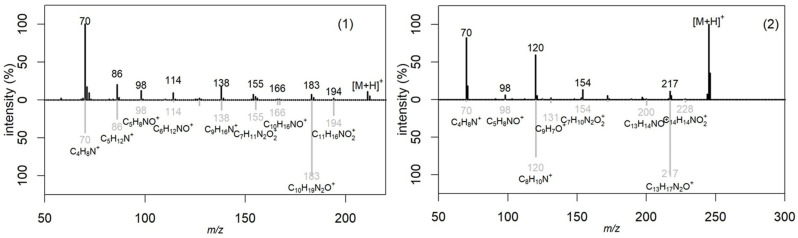
Comparison of fragmentation spectra of the precursor ion (black color) at *m*/*z* 211 (1) and *m*/*z* 245 (2) with mass spectra (gray color) from NIST database (NIST #1807166 and #1945836 from DB:hr_msms_nist, correspondingly).

The obtained metabolic profiles of the active extracts demonstrated both unique differences and partial overlaps. The chromatograms of the extracts of the *H. coralloides* 4 and *H. erinaceus* 1 strains did not have common peaks, which was manifested in a discrepancy in both the retention time and mass spectral characteristics (*m*/*z*), despite their taxonomic affiliation to the same genus. The fragment mass spectra for the precursor ions at *m*/*z* 211 (retention time 3.9 min; detected in *F. betulina* 3 and *F. pinicola* 2) and at *m*/*z* 245 (retention time 4.2 min; detected in *F. pinicola* 2 and *L. sulphureus* 4) were matched to the MS/MS spectra of cyclo(leucyl-prolyl) and cyclo(phenylalanyl-prolyl), respectively, from the NIST database (entries #1807166 and #1945836, DB: hr_msms_nist; Figure 4). Key fragment ions supported the identifications: *m*/*z* 70 was assigned to the dihydro-pyrrolium ion (C_4_H_8_N^+^), and *m*/*z* 90 was attributed to the prolyl ion (C_5_H_8_NO^+^). Furthermore, a neutral loss of carbon monoxide (CO, 28 Da) was observed, yielding the fragment ions at *m*/*z* 183 (C_10_H_19_N_2_O^+^) for cyclo(leucyl-prolyl) and *m*/*z* 217 (C_13_H_17_N_2_O^+^) for cyclo(phenylalanyl-prolyl).

Extracts of *F. pinicola* 2, *H. erinaceus* 1, and *L. sulphureus* 4 culture liquids also contained a compound characterized on chromatograms by a single peak with intense fluorescence, with a retention time of 4.8 min. However, this compound could not be detected by either mass spectrometric or diode array detection. This can be explained by the absence of ionizable functional groups (e.g., amino, carboxyl, or hydroxyl) and chromophore groups (such as a conjugated aromatic system).

A comparison between databases made it possible to identify individual metabolites. The metabolic profiles shown on the chromatograms demonstrated both unique differences and partial coincidences with previously studied fungi of the corresponding species. In particular, for the *H. erinaceus* 1 sample, a metabolite with a molecular weight ([M+H]^+^, *m*/*z* 420, peak 3) corresponding to the previously characterized isoindolinone, hericerin [32], was tentative detected, which exhibits cytotoxic activity against the human hepatocellular carcinoma cell line HepG2 [33]. Similarly, in profile, *L. sulphureus* 4 was found to match in weight ([M+H]^+^, *m*/*z* 255, peak 7) with the well-known sesquiterpenoid, sulfureuine H, previously isolated from cultures of the fungus *L. sulphureus* [34]. No metabolites were detected for the *H. coralloides* 4 sample that matched the mass of any previously known compounds for this species, such as corallocins [35].

## 3. Discussion

### 3.1. Results Discussion

Basidial fungi are a promising but insufficiently studied source of biologically active substances, in particular, antimicrobial compounds [10,36]. The work carried out has shown that many strains of basidiomycetes are capable of forming antimicrobial metabolites. Of the 18 strains we studied, 16 were capable of synthesizing antibacterial and/or antifungal metabolites, which accounted for about 89% of the tested sample of strains. Antimicrobial activity was shown by 16 extracts of culture liquids and 4 extracts of submerged mycelia. Thus, most of the target compounds belonged to exometabolites and were released by cells into the environment, which is consistent with the protective function of antimicrobial compounds.

The antibacterial activity of the extracts was observed more often than the antifungal activity. Twenty extracts were active against bacteria; twelve extracts were active against fungal test objects. The antibacterial activity was mainly directed against Gram-positive bacteria (20 active extracts), and much less often against Gram-negative bacteria (10 active extracts). The antifungal activity mainly consisted in suppressing the growth of mycelial cultures (*A. niger* and *F. oxysporum*); only 1 extract had activity against *C. albicans*.

Extracts of strains of representatives of the Agaricales order had the least antimicrobial activity. Of the 12 extracts studied, 7 showed neither antibacterial nor antifungal properties. The only representative of this order in our work that was promising for further work was the *A. borealis* strain 1. First, the extract of its culture liquid was active against Gram-positive bacteria (including *S. aureus*) and against the fungus *C. albicans*. Secondly, there are not enough studies on *A. borealis* [37,38]. In general, there is insufficient information about the antimicrobial properties’ genus Armillaria metabolites; it has been shown that extracts of *A. mellea* fruit bodies had weak antibacterial and antifungal activity [39].

Strains of the orders Polyporales and Russolales had a higher activity in comparison with strains of the order Agaricales. Strains of species from the order Polyporales showed activity, and four extracts of culture liquids were highly active against Gram-positive bacteria. Extracts of the *F. fomentarius* 1, *F. betulina* 3, and *F. pinicola* 2 culture liquids had high activity against *P. aeruginosa*. The mycelium extracts of these strains also had little antibacterial activity, unlike others mycelium extracts. MIC determination had shown that the culture liquid extracts of *F. betulina* 3, *F. pinicola* 2 and *L. sulphureus* 4 are more promising. The first two listed had a wide spectrum of action and MIC values of 640 µg/mL.

Currently, researchers are continuing to study the properties of *F. betulina* metabolites from the fruiting bodies, mycelium, and submerged culture. By 2024, more than 109 secondary metabolites of this basidiomycete had been isolated: 13 of them had antimicrobial activity, most of them were isolated from fruiting bodies [40]. For example, the compounds polyporenic acid C and t-cadinol have activity against various Gram-positive bacteria (*S. aureus*, *B. subtilis*, and *Mycobacterium phlei*) [41,42]. Piptamine had an MIC of 0.78 µg/mL against *S.aureus* and an MIC of 1.56 µg/mL against *Enterococcus faecalis* [43]. 1-epi-cubenol was active against the phytopathogens *Rhizoctonia solani* and *Choanephora cucurbitarum* [44,45]. We are not aware of any work with the same amount of activity of *F. betulina* culture liquid extracts against Gram-negative bacteria as was obtained in our study.

*F. pinicola* metabolites with antimicrobial and antitumor properties are known [46,47]. In addition to the frequently occurring activity against Gram-positive bacteria, studies have shown the activity of *F. pinicola* extracts against Gram-negative bacteria (*E. coli*, *P. aeruginosa*, *Salmonella typhimurium*, *K. pneumoniae*, and *A. baumanii*) and fungi, including dermatophytes (*Chrysosporium keratinophilum*, *Microsporum gypseum*, *Trichophyton terrestre*, and *Penicillium* species) [48,49]. Antimicrobial compounds from *F. pinicola* can be given 3-oxo-24-methyl-5a-lanost-8,25-dien-21-oic acid, 3-oxo-5α-lanost-8,24-dien-oic acid, and one ergostane in nature steroid, 22E-5α-ergosta7,9(11),22-trien-3b-ol, which are characterized by activity against *B. cerreus* with MICs of 32, 16, and 64 µg/mL, respectively [50]. Our work also showed activity against Gram-positive and Gram-negative bacteria, but the *F. pinicola* 2 extracts did not have activity on fungal test cultures.

The extract of the culture liquid *L. sulphureus* 4 had an MIC of 320 µg/mL against the tested bacteria of the genus *Staphylococcus*, which suggests the action selectivity of this strain of antimicrobial metabolites. More than 80 low-molecular-weight metabolites were isolated from *L. sulphureus*; antimicrobial activity was found in 12 individual compounds. Most of these compounds, for example, sulfureuine A, sulfureuine B, and laetiporin C, have shown antifungal activity against both mycelial and yeast-like pathogens [51]. In our work, no antifungal activity was detected in extracts of *L. sulphureus* 4.

The Russulales order in our study was represented by strains of two species, *H. erinaceus* and *H. coralloides*. Extracts of five out of six strains showed sufficiently high activity against Gram-positive bacteria; four strains suppressed the growth of mycelial fungi. In our study, the basidiomycete extract showed that the MIC values of the *H. erinaceus* 1 culture liquid extract against *S. epidermidis*, *S. haemoliticus*, *E. faecium,* and *A. baumanii* were 320 µg/mL. *H. erinaceus* metabolites were known to have antimicrobial activity. 1-(5-chloro-2-hydroxyphenyl)-3-methyl-1-butanone and 2,5-bis(methoxycarbonyl)terephthalic acid were active against *Helicobacter pylori* with MIC values of 12.5–50 µg/mL and 6.25–25 µg/mL, respectively [52]. Erinacine B, a neuroactive metabolite of *H. erinaceus*, has been found to be active against the bacterium *Bacillus atrophaeus* (MIC 2.5 µg/mL) and *B. subtilis* (MIC 5 µg/mL), as well as the fungi *Bortys cinerea* (MIC 10 µg/mL) and *R. solani* (MIC 20 µg/mL) [15]. Extracts of *H. erinaceus* were active against the bacteria *Proteus mirabillis*, *S. aureus*, and *P. aeruginosa* [53,54].

Among the studied strains, the leading producer was *H. corraloides* 4, whose culture liquid extract had high antibacterial and antifungal activities. Moreover, the mycelium extract of this strain was the only one of all mycelial extracts that showed high activity against Gram-positive bacteria and weak activity against fungi. The MIC values of the culture liquid extract of this strain ranged from 80 to 320 µg/mL against all tested strains of Gram-positive bacteria. Activity against clinical strains has been noted: against *S epidermidis* and *S. haemoliticus* (MIC 160 µg/mL), as well as against *E. faecium* resistant to vancomycin A (MIC 320 µg/mL). Unlike *H. erinaceus*, *H. coralloides* has been studied much less. The antibacterial activity of *H. coralloides* extracts against *S. aureus* is known [55,56].

No peaks with the same yield time and weights were observed in the chromatograms of the *H. coralloides* 4 and *H. erinaceus* 1 culture liquid extracts. The composition of the extracts of the *F. betulina* 3 and *F. pinicola* 2 culture liquids, on the contrary, had significant similarities; peaks with retention times of 1.7, 2.2, 3.5, and 3.9 min were present in both chromatograms.

HPLC-MS extracts of *F. pinicola* 2 and *L. sulphureus* 4 culture liquids reached a peak at 4.2 min with a fragment mass of 245 *m*/*z*. The chromatograms of the *F. pinicola* 2, *H. erinaceus* 1, and *L. sulphureus* 4 culture liquid extracts showed a peak with a retention time of 4.8 min. The observed coincidences show that extracts of representatives of the order Polyporales may contain similar metabolites not only within the same genus (in our work, the genus Fomitopsis), but also outside the genera, as in the case of coincidences between peaks in the extracts of *F. pinicola* 2 and *L. sulphureus* 4.

Using HPLC-MS, two metabolites were preliminary identified: hericerin in *H. erinaceus* 1 and sulfureuine H in *L. sulphureus* 4. The results suggested the presence of cyclo(leucyl-prolyl) in extracts of *F. betulina* 3 and *F. pinicola* 2, and cyclo(phenylalanyl-prolyl) in extracts of *F. pinicola* 2 and *L. sulfureus* 4.

Cyclo(phenylalanyl-prolyl) is a secondary metabolite produced by certain fungi and bacteria [57,58,59,60]. In the amphibian pathogen *Batrachochytrium dendrobatidis*, it functions as a secreted virulence factor [57]. Its impacts on host organisms include inducing melanization and disrupting cocoon development, as demonstrated in wax moth larvae, a model system for fungal pathogenesis. Furthermore, specific stereoisomers of cPP exhibit antifungal properties, suggesting their potential as biocontrol agents against fungal diseases [58,59,60].

Cyclo(leucyl-prolyl) is produced by microorganisms such as *Achromobacter xylosoxidans* [61] and *Bacillus amyloliquefaciens* [62], and exhibits significant antifungal activity. It acts primarily by inhibiting aflatoxin production in *Aspergillus* species like *A. parasiticus* and *A. flavus*, with direct fungicidal effects observed at higher concentrations.

Chromatography data obtained using various detectors, such as diode-matrix, fluorescent, and mass spectrometric detectors, demonstrate that selectivity with respect to the components of the mixture manifests itself in different ways, since each detection method is based on the unique physicochemical properties of molecules—the absorption of light at certain wavelengths, fluorescence ability, or ratio mass to charge, which makes it possible to selectively detect and identify substances that are invisible to other types of detectors.

### 3.2. Future Work

Since this study focused on selecting strains producing antimicrobial compounds, the next step involves a detailed analysis of their metabolic composition. The next step will involve fractionating the culture liquid extracts of the active strains, screening the fractions for antimicrobial activity, and, subsequently, isolating individual compounds from the active fractions. The isolated metabolites will be characterized using physicochemical methods, such as mass spectrometry and NMR spectroscopy. Additionally, the MICs of these purified metabolites will be determined and compared to the MICs of known natural antimicrobial metabolites.

## 4. Materials and Methods

### 4.1. Basidiomycetes Cultures

The following basidiomycete strains from the collection of the Laboratory of Biosynthesis of Biologically Active Compounds were used in the work: *Armillaria borealis* 1, *Fomes fomentarius* 1, *Fomitopsis betulina* 3, *Fomitopsis pinicola* 2, *Grifola frondose* 9, *Hericium coralloides* 1, *Hericium coralloides* 4, *Hericium coralloides* 18, *Hericium erinaceus* 1, *Hericium erinaceus* 9, *Hericium erinaceus* 17, *Hypsizygus marmoreus* 1, *Hypsizigus ulmarius* 1, *Laetiporus sulphureus* 4*, Pleurotus eryngii* 29, *Pleurotus ostreatus* 10, *Pleurotus pulmonarius* 1, and *Trametes versicolor* 1.

### 4.2. Sequencing

Genomic DNA was extracted from mycelial cultures using the CTAB buffer method [63]. The sequencing of the ITS rDNA (the internal transcribed spacer ITS1 and ITS2) was performed for taxonomic identification. Samples were amplified using the primers ITS1P and ITS4. The polymerase chain reaction was performed on a Techne TC412 amplifier (Barloworld Scientific, London, UK). The agarose gel electrophoresis method followed by spectrophotometric analysis (Nanodrop, Madison, WI, USA) was used to control the quality of the work performed. Sequencing using the Sanger method with dideoxynucleoside triphosphates was performed on an Applied Biosystems 3130 sequencer (Applied Biosystems, Waltham, MA, USA). The alignment of the obtained sequences was performed using the Clustal W program (MEGA version 6.0 software package). Sequence analysis was conducted using the BLAST version 2.1.2 algorithm against the NCBI GenBank database, with species-level identification defined as ≥99.5% sequence similarity to validated reference sequences.

### 4.3. Submerged Cultivation of Basidiomycetes Strains

The basidiomycete strains were grown on a solid PDA medium in a thermostat at 26 °C until the culture had completely mastered the surface of the nutrient agar. Storage was carried out at 4 °C. Submerged cultivation was carried out in two stages, the first of which produced liquid inoculum, the second a submerged culture for further extraction. The composition of the first medium was given in [25,64]. The pH value of second media before sterilization was 6.1. The pH value of the culture liquid was measured using a pH meter PHS-3C (Yoke, Shanghai, China). Submerged cultivation was carried out at 25 °C on an orbital shaker (Infors RC-406, Bottmingen, Switzerland) at 200 rpm. The liquid seed mycelium was grown for 6 days; the duration of fermentation itself ranged from 5–7 days, depending on the strain. The volume of liquid inoculum used was 10%. The mycelium was separated from the culture liquid by centrifugation (Laboratory centrifuge UC-1536E, ULAB, Nanjing, China) or by filtration. The submerged mycelia were lyophilized (LS-500 freeze dryer, Prontech LLC, Moscow, Russia) and dry mass yield was determined gravimetrically.

### 4.4. Extraction of Culture Liquids and Submerged Mycelia

Extraction of the culture liquid was carried out with EtOAc (Vecton, Saint-Petersburg, Russia) as extractant. After lyophilization and homogenization the submerged mycelium was extracted with 95% EtOH (1/10 *w*/*v*) for 15 min on a magnetic stirrer (RITM-01, Expert, Russia) under constant agitation. After all the steps, both extracts were evaporated at 40 °C using a KNF RC600 rotary evaporator (KNF, Freiburg im Breisgau, Germany).

To study antimicrobial properties by agar well diffusion method, dry extracts were dissolved in 25% EtOH to obtain a solution with a concentration of 10 mg/mL. To determine the minimum inhibitory concentration of the most active extracts, the extracts were dissolved in 5% DMSO (Vecton, Saint-Petersburg, Russia).

### 4.5. Agar Well Diffusion Method

Strains of bacteria and fungi were used as test objects. These included Gram-positive bacteria: *Bacillus subtilis* ATCC 6633, *Micrococcus luteus* ATCC 9341, and *Staphylococcus aureus* ATCC 43300; Gram-negative bacterium *Pseudomonas aeruginosa* ATCC 27853; and fungi: *Candida albicans* ATCC 14053, *Aspergillus niger* ATCC 16404, and *Fusarium oxysporum* VKPM F-890.

Bacterial strains were cultured on Mueller–Hinton agar (AcuMedia, San Bernardino, CA, USA), while fungal strains were grown on the same medium supplemented with 2% glucose, following the protocol described in [65]. Prior to inoculation, microbial suspensions were adjusted to 0.5 McFarland standard using a DEN-1 densitometer (Bio-San, Riga, Republic of Latvia), and were evenly spread on the surface of the agar medium in Petri dishes.

Wells with a diameter of 9 mm were used, into which 100 µL of the prepared working solution of basidiomycete extract were added; 25% EtOH was used as a control. Incubation was carried out for 20–24 h at 28 °C for *F. oxysporum* and at 35 °C for the other test organisms. The experiments were carried out three times.

### 4.6. Minimum Inhibitory Concentration (MIC) Determination

To carry out the analysis by microtitre broth dilution method, following strains of test organisms were used: Gram-positive bacteria *S. aureus* ATCC 25923, *S. aureus* 10, *Staphylococcus epidermidis* 533, *Staphylococcus haemoliticus* 585, and *Enterococcus faecium* 569; and Gram-negative bacteria *Acinetobacter baumanii* ATCC 5696, *Escherichia coli* ATCC 25922, *Klebsiella pneumoniae* ATCC 13883, *Salmonella cholerasuis* ATCC 14028, and *Proteus vulgaris* ATCC 13315.

The sample dilutions from 1280 to 40 µg/mL were used as a modification of the method for extracts. A suspension of microorganisms was prepared in a sterile isotonic sodium chloride solution, bringing the inoculum density to 0.5 according to the McFarland standard (1.5 × 10^8^ CFU/mL). The resulting inoculum was diluted to a concentration of 5 × 10^5^ CFU/mL with Muller–Hinton broth. Levofloxacin (Belmedpreparaty RUP, Minsk, Belarus) was used as control antibiotic. Wells with Muller–Hilton broth and wells with bacteria without the addition of an antibiotic were used as controls. Incubation of the cultures in the tablet was carried out in a thermostat at a constant temperature of 36 °C for 24 h. The experiments were carried out three times.

### 4.7. High-Performance Liquid Chromatography with Tandem Mass Spectrometry (HPLC-MS)

HPLC was carried out with Agilent 1260 Infinity UPLC system (Agilent Technologies Inc., Santa Clara, CA, USA), that was coupled with diode array (G4212A), fluorescence (G1321A), and mass spectrometry (G1956B Single Quad LC/MSD SL and Triple Quad LC/MSD G6430A QqQ) detectors being used to study the composition of the extract.

Chromatography was performed on a Zorbax SB-C18 reversed-phase column (1.8 um particle size, 100 × 2.1 mm i.d.) at 30 °C in a gradient elution mode (90% A -> 10% A for 10 min). The components of the mobile phase were as follows: A—0.1% (by volume) aqueous formic acid solution, and B—acetonitrile. The flow rate was 0.150 mL/min. The analysis time was 20 min. The injection volume was 10 µL. The temperature of the column was 30 °C.

In routine analysis, mass spectrometric detection was performed under electrospray ionization (ESI) in the scanning mode using single quad detector (LC/MSD), with mass range from *m*/*z* 100 to 1000 positive and negative ions. A drying stream of nitrogen was supplied to the ionization source at a rate of 5 L/min and a temperature of 350 °C. The input capillary voltage was +4000 V (for positive ions). The Agilent ChemStation software (version 04.03) was used for registration, analysis, and processing of experimental data.

Peak identification was performed using tandem mass spectrometry (MS/MS) on a triple quadrupole LC/MS instrument. Precursor ions (selected based on data in Table 6) were fragmented with a collision energy of 10 V, and the resulting product ions were scanned from *m*/*z* 50 to 500. The acquired spectra were matched against the NIST23 tan-dem MS library using NIST Mass Spectral Search Program (version 2.4, build 25 March 2020). The data acquisition and processing were carried out using Agilent MassHunter Qualitative Analysis software (version B.07.00).

Spectroscopic detection was performed using a diode array detector monitoring wavelengths of 210, 230, 260, 280, and 351 nm, coupled with a fluorescence detector set to an excitation wavelength of 230 nm and an emission wavelength of 460 nm.

### 4.8. Statistical Analysis

All experiments on the strains’ submerged cultivation and determination of extracts antimicrobial activity were carried out in three repetitions. The results are presented as the mean and standard deviation. The results of the study were processed using the Microsoft Excel software package.

## 5. Conclusions

Basidiomycetes produce exo- and endometabolites with antimicrobial properties, which makes them promising sources of new active molecules. This work demonstrated the extracts’ antimicrobial activity of basidiomycetes from the orders of Agaricales, Polyporales, and Russolales. Of the 18 strains, 16 showed activity. Strains of the orders Polyporales and Russulales had a higher activity in comparison with strains of the order Agaricales. As a rule, the activity of exometabolites was higher than the activity of endometabolites; this was shown when comparing the activity of mycelium extracts and culture liquids. Antibacterial activity against Gram-positive bacteria was more common (for 20 extracts out of 36). The extract of the *H. coralloides* 4 culture liquid had the highest activity based on the results of the MIC determination. Extracts of *H. erinaceus* 1, *L. sulphureus* 4, *F. betulina* 3, and *F. pinicola* 2 cultural liquids also showed high activity. Extracts of these strains were analyzed by the HPLC-MS method. This analysis revealed the potential presence of metabolites: hericerin in *H. erinaceus* 1, and sulfureuine H in *L. sulphureus* 4. It was also shown that *H. erinaceus* 1 and *H. coralloides* 4 did not contain the same metabolites, and *F. betulina* 3 and *F. pinicola* 2 contained several identical metabolites, in particular, leucilprolil.

Thus, as a result of the study, taxonomic groups were identified whose representatives are promising for screening antimicrobial metabolites, knowledge about the biosynthetic capabilities of *H. coralloides* 4 and *A. borealis* 1 was expanded, and the leading strains *H. coralloides* 4, *H. erinaceus* 1, *L. sulphureus* 4, *F. betulina* 3, and *F. pinicola* 2 were selected for further physicochemical and medical and biological research.

## Figures and Tables

**Table 1:** Submerged cultivation characteristics of the studied basidiomycetes strains.

Strain	Cultivation Day	Culture Liquid pH	Dry Biomass Yield, g/L	Strain	Cultivation Day	Culture Liquid pH	Dry Biomass Yield, g/L
*Armillaria borealis* 1	6	4.67 ± 0.23	3.26 ± 0.14	*Hericium erinaceus* 9	7	4.62 ± 0.16	2.77 ± 0.08
*Fomes fomentarius* 1	5	2.63 ± 0.15	4.14 ± 0.13	*Hericium erinaceus* 17	6	4.30 ± 0.05	9.41 ± 0.21
*Fomitopsis betulina* 3	5	1.34 ± 0.02	3.83 ± 0.03	*Hypsizygus marmoreus* 1	5	5.28 ± 0.12	6.75 ± 0.07
*Fomitopsis pinicola* 2	6	2.87 ± 0.23	3.28 ± 0.08	*Hypsizygus ulmarius* 1	5	6.54 ± 0.06	11.92 ± 0.06
*Grifola frondosa* 9	5	5.36 ± 0.12	1.40 ± 0.15	*Laetiporus sulphureus* 4	6	2.55 ± 0.24	1.14 ± 0.25
*Hericium coralloides* 1	6	4.91 ± 0.15	5.98 ± 0.12	*Pleurotus eryngii* 29	6	4.23 ± 0.13	6.45 ± 0.14
*Hericium coralloides* 4	7	4.56 ± 0.04	6.14 ± 0.46	*Pleurotus ostreatus* Ls	6	4.69 ± 0.15	5.36 ± 0.09
*Hericium coralloides* 18	7	4.55 ± 0.04	8.79 ± 0.17	*Pleurotus pulmonarius* 1	5	5.13 ± 0.14	13.31 ± 0.06
*Hericium erinaceus* 1	6	4.96 ± 0.32	6.23 ± 0.23	*Trametes versicolor* 1	6	5.15 ± 0.15	1.27 ± 0.11

**Table 2:** Antimicrobial activity of basidiomycetes extracts. Order Agaricales.

Strain	Extract	Inhibition Zone, mm						
		*S. aureus* ATCC 43300	*M. luteus* ATCC 9341	*B. subtilis* ATCC 6633	*P. aeruginosa* ATCC 27853	*C. albicans* ATCC 14053	*F. oxysporum* VKPM F-890	*A. niger* ATCC 16404
*Armillaria borealis* 1	Cul. liquid *	**15.0 ± 1.0**	12.3 ± 0.6	12.7 ± 1.2	/ **	12.7 ± 0.6	11.3 ± 1.5	/
	Mycelium	/	/	/	/	/	/	/
*Hypsizygus marmoreus* 1	Cul. liquid	/	/	14.7 ± 1.2	/	/	15.3 ± 1.1	/
	Mycelium	/	/	/	/	/	/	/
*Hypsizygus ulmarius* 1	Cul. liquid	/	**16.3 ± 1.2**	13.7 ± 0.6	14.3 ± 0.6	/	13.0 ± 1.0	/
	Mycelium	/	/	/	/	/	/	/
*Pleurotus eryngii* 29	Cul. liquid	/	/	12.7 ± 0.6	/	/	12.3 ± 1.5	/
	Mycelium	/	/	/	/	/	/	/
*Pleurotus ostreatus Ls*	Cul. liquid	12.0 ± 1.0	**15.7 ± 1.5**	/	/	/	13.3 ± 0.6	/
	Mycelium	/	/	/	/	/	/	/
*Pleurotus pulmonarius* 1	Cul. liquid	/	/	/	/	/	/	/
	Mycelium	/	/	/	/	/	/	/

* Cultural liquid; ** inactive. The values of growth inhibition zones above 15 mm are highlighted in bold.

**Table 3:** Antimicrobial activity of basidiomycetes extracts. Order Polyporales.

Strain	Extract	Inhibition Zone, mm						
		*S. aureus* ATCC 43300	*M. luteus* ATCC 9341	*B. subtilis* ATCC 6633	*P. aeruginosa* ATCC 27853	*C. albicans* ATCC 14053	*F. oxysporum* VKPM F-890	*A. niger* ATCC 16404
*Fomes fomentarius* 1	Cul. liquid *	**21.3 ± 1.5**	**27.7 ± 1.1**	**16.7 ± 0.6**	**21.7 ± 1.5**	/ **	/	/
	Mycelium	12.7 ± 0.6	**15.3 ± 2.5**	14.0 ± 1.0	11.3 ± 1.5	/	/	/
*Fomitopsis betulina* 3	Cul. liquid	**29.0 ± 1.0**	**30.3 ± 1.5**	**21.7 ± 1.5**	**23.7 ± 0.6**	/	13.7 ± 0.6	/
	Mycelium	14.7 ± 1.5	**18.3 ± 0.6**	**15.0 ± 1.0**	13.0 ± 1.7	/	/	/
*Fomitopsis pinicola* 2	Cul. liquid	**35.7 ± 1.7**	**33.3 ± 2.1**	**23.0 ± 0.0**	**25.7 ± 1.5**	/	13.0 ± 1.0	/
	Mycelium	/	11.0 ± 1.0	12.3 ± 1.3	/	/	/	/
*Grifola frondosa* 9	Cul. liquid	10.3 ± 0.7	13.3 ± 1.5	12.0 ± 2.0	12.0 ± 1.0	/	/	/
	Mycelium	/	/	/	/	/	/	/
*Laetiporus sulphureus* 4	Cul. liquid	**33.7 ± 1.3**	**25.3 ± 1.3**	**26.7 ± 1.5**	**15.5 ± 2.5**	/	/	/
	Mycelium	/	/	/	/	/	/	/
*Trametes versicolor* 1	Cul. liquid	12.0 ± 2.0	11.3 ± 0.7	11.3 ± 0.7	/	/	/	/
	Mycelium	/	/	/	/	/	/	/

* Cultural liquid; ** inactive. The values of growth inhibition zones above 15 mm are highlighted in bold.

**Table 4:** Antimicrobial activity of basidiomycetes extracts. Order Russulales.

Strain	Extract	Inhibition Zone, mm						
		*S. aureus* ATCC 43300	*M. luteus* ATCC 9341	*B. subtilis* ATCC 6633	*P. aeruginosa* ATCC 27853	*C. albicans* ATCC 14053	*F. oxysporum* VKPM F-890	*A. niger* ATCC 16404
*Hericium coralloides* 1	Cul. liquid *	**24.0 ± 1.0**	**18.3 ± 0.7**	**20.7 ± 1.1**	/ **	/	12.0 ± 2.0	11.7 ± 0.3
	Mycelium	/	/	/	/	/	/	/
*Hericium coralloides* 4	Cul. liquid	**28.3 ± 2.1**	**26.6 ± 0.3**	**26.0 ± 1.0**	12.7 ± 1.1	/	**15.0 ± 2.0**	13.3 ± 1.5
	Mycelium	**25.0 ± 1.0**	**25.7 ± 0.8**	**21.7 ± 2.1**	/	/	11.0 ± 1.0	11.0 ± 2.0
*Hericium coralloides* 18	Cul. liquid	**23.7 ± 0.7**	**18.0 ± 2.0**	**17.3 ± 1.5**	13.0 ± 1.0	/	13.7 ± 0.3	/
	Mycelium	/	/	/	/	/	/	/
*Hericium erinaceus* 1	Cul. liquid	**20.6 ± 1.5**	14.7 ± 0.3	13.7 ± 1.5	/	/	**15.0 ± 1.0**	10.7 ± 1.5
	Mycelium	/	/	/	/	/	/	/
*Hericium erinaceus* 9	Cul. liquid	/	/	/	/	/	/	/
	Mycelium	/	/	/	/	/	/	/
*Hericium erinaceus* 17	Cul. liquid	**20.0 ± 2.0**	**18.3 ± 0.7**	12.0 ± 1.0	/	/	/	/
	Mycelium	/	/	/	/	/	/	/

* Cultural liquid; ** inactive. The values of growth inhibition zones above 15 mm are highlighted in bold.

**Table 5:** Minimal inhibitory concentrations of cultural liquid extracts of promising basidiomycete strains.

Test Cultures	MIC, µg/mL					
	*Fomes fomentarius* 1	*Fomitopsis betulina* 3	*Fomitopsis pinicola* 2	*Hericium erinaceus* 1	*Hericium coralloides* 4	*Laetiporus sulphureus* 4
**Gram-positive bacteria**						
*Staphylococcus aureus* 25923 ATCC	>1280	640	640	640	80	320
*Staphylococcus aureus* 10 *	>1280	640	1280	640	320	320
*Staphylococcus epidermidis* 533 *	640	640	640	320	160	320
*Staphylococcus haemoliticus* 585 *	640	640	640	320	160	320
*Enterococcus faecium* 569 *	1280	1280	1280	320	320	640
**Gram-positive bacteria**						
*Acinetobacter baumanii* 5696 ATCC	640	640	640	320	>1280	640
*Escherichia coli* 25922 ATCC	640	640	640	640	>1280	1280
*Klebsiella pneumoniae* 13883 ATCC	>1280	640	640	640	>1280	1280
*Salmonella cholerasuis* 14028 ATCC	>1280	640	>1280	640	>1280	1280
*Proteus vulgaris* 13315 ATCC	>1280	1280	1280	640	640	640

* clinical strain. Antimicrobial activity is color-coded as follows: red (significant), orange (moderate), green (weak/close to threshold).

**Table 6:** Most relevant HPLC-MS data for basidiomycetes culture liquid extracts.

Basidiomycete strain: *Fomitopsis betulina* 3						
Peak	RT, min	UV absorbance: RT min, %		Fluorescence: RT min, mAU em. 230 nm exc. 460 nm	MS data, EIC: *m*/*z* (intensity at RT, %)	
		280 nm	210 nm		MS (+)	MS (−)
1	1.8	1.8 (100%)	1.7 (67%)			
2	2.2	2.2 (47%)	-		225; 227	
3	3.5	-	3.5 (33%)	3.6 (100%)		303; 325
4	3.9				211	
5	10.5	-	10.5 (68%)	-	-	527
Basidiomycete strain: *Fomitopsis pinicola* 2						
Peak	RT, min	UV absorbance: RT min, %		Fluorescence: RT min, mAU em. 230 nm exc. 460 nm	MS data, EIC: *m*/*z* (intensity at RT, %)	
		280 nm	210 nm		MS (+)	MS (−)
1	1.7		1.7 (67%)			201; 313
2	2.2	2.2 (100%)			225; 227	
3	3.5		3.5 (73%)		277	303; 393
4	3.9				211	
5	4.2	4.2 (27%)			245	
6	4.8			4.8 (100%)	267; 359	
Basidiomycete strain: *Laetiporus sulphureus* 4						
Peak	RT, min	UV absorbance: RT min, %		Fluorescence: RT min, mAU em. 230 nm exc. 460 nm	MS data, EIC: *m*/*z* (intensity at RT, %)	
		280 nm	210 nm		MS (+)	MS (−)
1	4.2	4.2 (100%)			245	269
2	4.8			4.8 (100%)		
3	6	5.1	5.1 (65%)			
4	7	5.5	5.5 (37%)			255
5	10.5	10.5 (100%)			280	
Basidiomycete strain: *Hericium coralloides* 4						
Peak	RT, min	UV absorbance: RT min, %		Fluorescence: RT min, mAU em. 230 nm exc. 460 nm	MS data, EIC: *m*/*z* (intensity at RT, %)	
		280 nm	210 nm		MS (+)	MS (−)
1	1.7	1.7 (100%)				225
2	4.6	4.6 (82%)				
3	8.0	8.0 (74%)				564
4	9.5		9.5 (83%)		256; 511	540
5	10.5		10.5 (100%)		282; 565	
Basidiomycete strain: *Hericium erinaceus* 1						
Peak	RT, min	UV absorbance: RT min, %		Fluorescence: RT min, mAU em. 230 nm exc. 460 nm	MS data, EIC: *m*/*z* (intensity at RT, %)	
		280 nm	210 nm		MS (+)	MS (−)
1	3.7			3.5(26%)	246; 476	227; 455
2	4.5	4.5 (24%)			378; 420	376; 418
3	4.8			4.8(16%)		
4	5.7	5.7 (100%)				293; 587
5	11.1	11.1 (66%)		11.3(100%)	280; 296	358

## Data Availability

The original data are available from the corresponding author upon request.

## Abbreviations

The following abbreviations are used in this manuscript:

DNA	Deoxyribonucleic acid
DMSO	Dimethyl sulfoxide
HPLC-MS	High-performance liquid chromatography–mass spectrometry
MIC	Minimum inhibitory concentration
PDA	Potato dextrose agar
RT	Retention time
UV	Ultraviolet
